# A rapid and quantitative technique for assessing IgG monomeric purity, calibrated with the NISTmAb reference material

**DOI:** 10.1007/s00216-019-02029-0

**Published:** 2019-08-02

**Authors:** Peter P. Reader, Rouslan V. Olkhov, Shaun Reeksting, Anneke Lubben, Christopher J. Hyde, Andrew M. Shaw

**Affiliations:** 1 University of Exeter Medical School, Heavitree Road, Exeter, EX1 2LU UK; 2 College of Life and Environmental Sciences, University of Exeter, Stocker Road, Exeter, EX4 4QD UK; 3 Chemical Characterisation and Analysis Facility, University of Bath, Claverton Down, Bath, BA2 7AY UK

**Keywords:** Biosensor, Antibody, Monomeric purity, NISTmAb, Immunoassay

## Abstract

The fraction of intact monomer in a sample (moles/moles), the monomeric purity, is measured as a quality control in therapeutic monoclonal antibodies but is often unknown in research samples and remains a major source of variation in quantitative antibody-based techniques such as immunoassay development. Here, we describe a novel multiplex technique for estimating the monomeric purity and antigen affinity of research grade antibody samples. Light scattering was used to simultaneously observe the mass of antibody binding to biosensor surfaces functionalised with antigen (revealing Fab binding kinetics) or protein A/G (PAG). Initial estimates of monomeric purity in 7 antibody samples including a therapeutic infliximab biosimilar were estimated by observing a mass deficit on the PAG surface compared to the NISTmAb standard of high monomeric purity. Monomeric purity estimates were improved in a second step by observing the mass of antigen binding to the mass of antibody on the PAG surface. The NISTmAb and infliximab biosimilar displayed tightly controlled stoichiometries for antigen binding of 1.31 ± 0.57 and 1.71 ± 0.16 (95% confidence interval)—within the theoretical limit of 1–2 antigens per antibody depending on avidity. The other antibodies in the panel displayed antigen binding stoichiometries in the range 0.06–1.15, attributed to lower monomeric purity. The monomeric purity estimates were verified by electrospray ionization mass spectrometry (ESI), the gold standard technique for structural characterization of antibodies. ESI data indicated that the NISTmAb and infliximab biosimilar samples had monomeric purity values of 93.5% and 94.7%, respectively, whilst the research grade samples were significantly lower (54–89%). Our results demonstrate rapid quality control testing for monomeric purity of antibody samples (< 15 min) which could improve the reproducibility of antibody-based experiments.

**Electronic supplementary material:**

The online version of this article (10.1007/s00216-019-02029-0) contains supplementary material, which is available to authorized users.

## Introduction

In non-therapeutic antibodies used for research, a link has been made between poor reproducibility of results and variability in antibody quality [1–3]. Antibody samples are known to undergo degradation over time during storage [4] and in vivo and the fraction of intact monomer in a sample, the monomeric purity, is tested to ensure potency of therapeutic antibodies [5]. Antibodies for research should also be assessed for monomeric purity to ensure reproducible experiments [6] and a rapid technique for assessing the monomeric purity of antibody samples could test the extent of degradation in samples immediately before use.

Lot-to-lot comparisons of antibody samples should be performed with reference standards [7]. The National Institute of Standards and Technology (NIST) provides a monoclonal antibody, the NISTmAb, designed to support biopharmaceutical innovation by serving as reference standard for comparable evaluation of antibody-based techniques between different laboratories [8–10]. The NISTmAb is a recombinant humanized IgG1ĸ with a known sequence [11] specific to the respiratory syncytial virus protein F (RSVF) [12]. The NISTmAb is well characterized with a known monomeric purity variation of 96.7–98.7% between batches, as characterized by comparing the relative peak areas of a non-denaturing size exclusion chromatogram [8, 9]. The NIST 8671 certificate for the material used in the present study quotes a monomeric purity of 96.63 ± 0.46%.

**Fig. 1:**
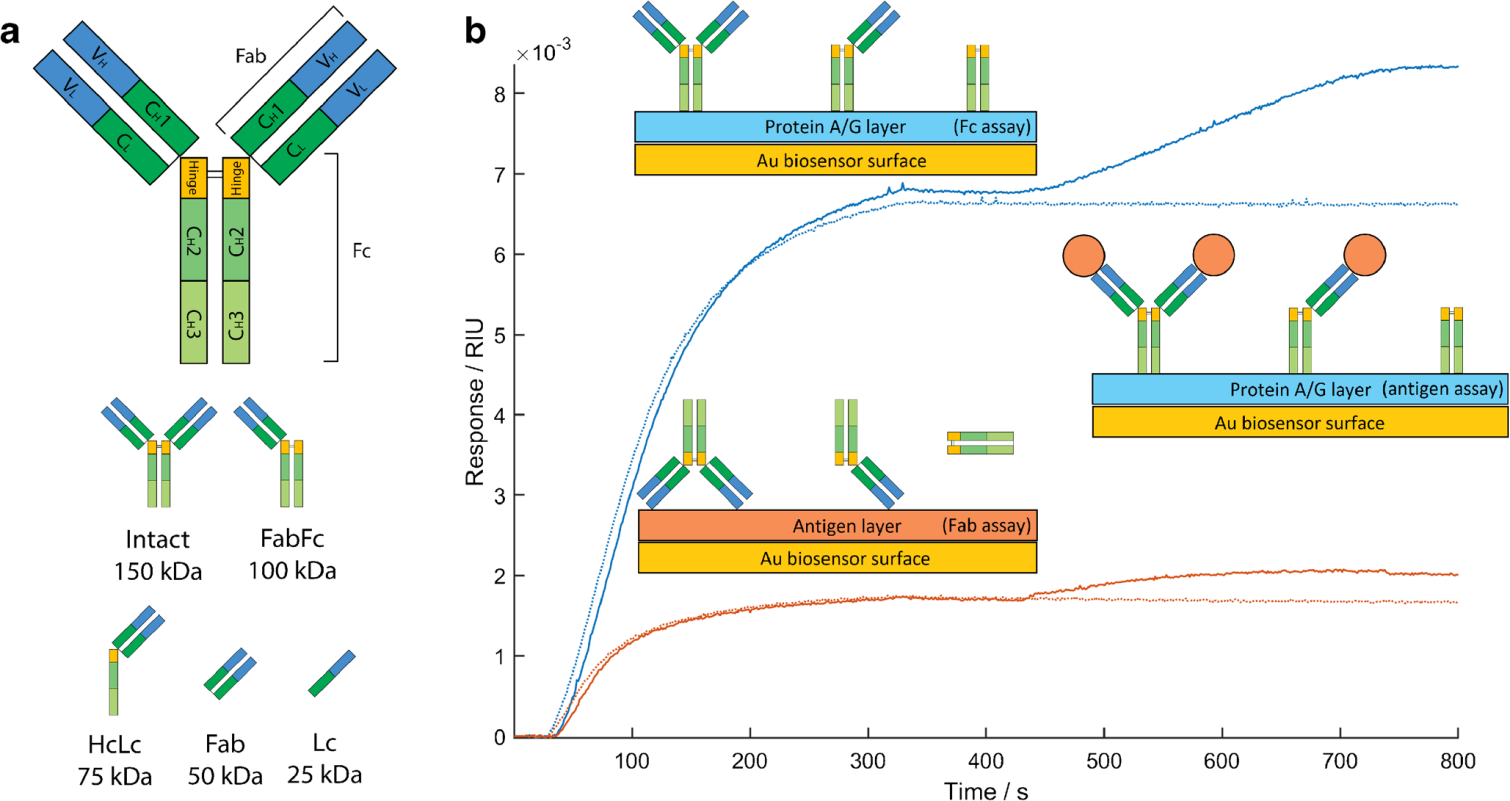
Schematic of fragmentation patterns of a typical IgG and their binding properties: **a** structure of IgG. Variable and constant domains are marked V and C with subscripts for light and heavy chain marked L or H. Heavy chain regions are numbered 1, 2, and 3 starting from the N-terminus and black lines indicate disulphide bonds. The most abundant antibody fragments recorded in IgG samples and their respective molecular masses are also shown. Heavy chain and light chain fragments are labelled Hc and Lc. The 50 kDa fragment may originate from the Fab region as shown or the Fc region. Similarly, the 25 kDa fragment may originate from the Lc as shown, the Hc of the Fab region, or Hc of the Fc region. **b** Biophotonic assays used to characterize the monomeric purity of an IgG sample. The Fab assay uses a sensor surface functionalised with antigen (orange) to confirm antibody specificity and derive antibody-antigen binding kinetics. The Fc assay uses a sensor surface functionalised with PAG (blue) to capture Fc fragments. Antibody captured by the Fc assay is used as an antigen assay to determine the antibody-antigen binding stoichiometry. Intact IgG may bind a maximum of 2 antigens (orange circles), whereas samples containing fragments lacking Fab regions will show a lack of antigen capture. The sensor response of the antigen surface (orange) and PAG surface (blue) to the NISTmAb is shown, with and without the inclusion of the antigen assay step (solid and dotted lines respectively) to highlight the signal of antigen capture

Fragmentation in monoclonal antibody samples is frequently observed at the hinge region disulphide bonds. In this region, a single polypeptide connects the Fab and Fc fragments and hence cleavage is followed by separation of these fragments [13]. The most abundant antibody fragments and their respective masses observed by non-denaturing size exclusion chromatography (SEC) [4] are shown in Fig. 1a.

SEC, sodium dodecyl sulphate-polyacrylamide gel electrophoresis (SDS-PAGE), and mass spectrometry (MS) are the gold standard techniques used for characterization and stability assessment of antibodies [4, 14, 15]. Non-reducing SDS-PAGE analysis of a papain digestion of the NISTmAb shows bands for Fab and Fc fragments at 50 kDa, whilst under reducing conditions, the disulphide bonds between heavy and light chains are separated to produce two species of 25 kDa [16]. The primary amino acid sequence and structure of antibodies are readily confirmed by liquid chromatography and mass spectrometry (LC-MS/MS) [17]; however, there are no easy methods to establish the monomeric purity or biological activity of a sample immediately before use. Therefore, in the current study, we consider the role of a multiplexed plasmonic biosensor-based assay for rapid monitoring of antibody monomeric purity in a research setting with similar analysis times to MS.

Plasmonic biosensors are typically used to observe antibody binding kinetics and are fundamentally mass sensors. Accurate kinetic parameters may be derived using antibody samples of high monomeric purity, careful tethering chemistry, and fitting of a mathematical kinetic model to multiple low-concentration interactions simultaneously in a global fit [18, 19]. However, kinetic analyses are fundamentally limited by unknown antibody concentration and monomeric purity. The presence of multiple binding interactions in fragmented antibody samples invalidates the assumed Langmuirian 1:1 binding interaction model which is commonly fit to plasmonic data [18].

In this paper, we report a novel multiplex technique to simultaneously assess antibody affinity and monomeric purity, based on our in-house biosensor platform [19, 20]. Sensor surfaces are functionalised with either antigen or the Fc binding protein A/G (PAG) to generate assays for Fab and Fc fragments. PAG has 3 sterically available binding sites for the Fc region of intact IgG and binds with high affinity to antibodies from several species [20]. The technique described here is comprised of two steps, shown in Fig. 1. In the first step, antibody binding to the Fab assay provides data for kinetic analysis of the antibody-antigen interaction and the mass of antibody binding to the Fc assay is recorded simultaneously. For a constant number of binding sites on the Fc assay surface, an initial estimate of monomeric purity may be obtained by observing a mass deficit in degraded samples compared to the NISTmAb. In the second step, the mass of antigen which can bind to the antibodies immobilized on the PAG surface (an antigen assay) is recorded. The stoichiometry of the antibody-antigen binding reaction on the PAG surface is derived from the masses of antibody and antigen, and samples with high monomeric purity should bind an average of 1–2 antigens per antibody, whereas antibody fragments lacking Fab regions should display a lower stoichiometry. A panel of eight antibody samples was tested: the NISTmAb, a therapeutic infliximab biosimilar, and six commercially available monoclonal and polyclonal samples for research use. Our results are validated with native ESI-quadrupole time-of-flight (QTOF) mass spectrometry—a gold standard technique for assessing the relative abundance of antibody fragmentation products in a sample [21].

## Materials and methods

Pierce recombinant protein A/G (#21186) and anti-C5a C17/5 (#GAU025-05-02) were obtained from Thermo Scientific. Bovine serum albumin (BSA) (#A7030) was obtained from Sigma-Aldrich. NIST RM 8671 was purchased from the National Institute of Standards and Technology and respiratory syncytial virus protein F (RSVF, #11049-V08B) was obtained from Sino Biological Inc. C5a protein (#A145) was purchased from Complement Technologies Inc. Anti-CRP monoclonal, anti-CRP polyclonal, anti-TBG, TNFα, CRP, and TBG were all obtained from a major research reagent provider. Infliximab was obtained from clinical storage, reflecting the conditions of therapeutic antibodies before use. Standard instrument running buffer and sample dilution buffer was phosphate-buffered saline (PBS) supplied in tablet form by Sigma-Aldrich. Ortho-phosphoric acid (85%) was obtained from Fluka and a 0.01 M aqueous solution used as regeneration buffer. The absorbance of the NISTmAb and other antibody samples was measured at 280 nm with a NanoDrop instrument and converted to total protein concentration using Beer’s Law and a molar extinction coefficient of 210,000 M^−1^ cm^−1^ (as recommended by the manufacturer).

The binding kinetics and monomeric purity of 8 antibody samples was assessed using our novel biophotonic multiplexed biosensor platform, described in detail elsewhere [19, 20, 22]. Briefly, gold nanoparticles are printed onto an aminated glass surface using an inkjet printer and the nanoparticles are chemically grown on the surface to ~60 nm in diameter. The gold nanoparticles are illuminated in a total internal reflection configuration with the localized nanoparticle plasmon scattering light normal to the surface depending on the refractive index (mass) of protein in the plasmon field. The scattered light intensity is monitored in real time using a video camera capturing data at 30 frames per second.

A multiplexed biophotonic array is fabricated by functionalising each of the 150 array spots with proteins using EDC/NHS coupling chemistry [22]. The array contains some reference spots functionalised with BSA which are used to correct for variations in light intensity, temperature, and non-specific binding during the time course of the experiment. Spots functionalised with antigen are used for specific antibody capture via the Fab region (a Fab assay) and spots functionalised with PAG are used for antibody capture via the Fc region (an Fc assay). PAG binds to IgG in an orientation presenting the Fab regions away from the sensor surface into the solution, as seen in affinity column chromatography. Once loaded with a given antibody, the PAG surface may be used to capture antigen from a sample (an antigen assay).

A multiplexed array was printed with the Fc and Fab assays: PAG and antigen were printed with 23 repeats each from 1 mg/mL solutions in PBS. The control assay elements are printed with 25 repeats from a 1 mg/mL solution of BSA in PBS. The responses of assay elements were averaged to improve the signal-to-noise ratio and eliminate the effects of any inhomogeneous protein tethering density. The array surface was blocked with BSA (1 mg/mL, 300 s) before use to minimize non-specific binding. The response of each assay to refractive index (RI) change was calibrated with a step change in running buffer from PBS to 2 × PBS concentration with a known bulk RI change of 1.6 mRIU. The mean limit of detection per individual sensor element was 6.6 (± 2.9) × 10^−2^ mRIU.

The Fc assay is calibrated on each array by injecting the NISTmAb at concentrations of 1.56, 3.23, and 6.25 nM and the response of the Fc and Fab assays recorded simultaneously. The data were used for kinetic analysis of the NISTmAb with a global fit to the Langmuir adsorption isotherm [19, 20]. The arrays were regenerated with a 0.01 M solution of phosphoric acid for 100 s between samples. The PAG surface has been demonstrated previously to be stable over > 20 regeneration cycles [20]. For monomeric purity testing, all antibody samples were diluted to 100 nM (based on manufacturer stated concentration) and injected over the assay surface for a fixed period of 300 s to monitor association kinetics to the Fab assay and Fc assay. The assays were washed in PBS running buffer for 500 s to monitor dissociation kinetics. Each antigen sample was prepared at 100 nM in PBS and injected over the assay surfaces for 300 s to observe antigen capture by IgG previously loaded on the PAG surface.

The sensor response to antibody or antigen binding at maximum surface coverage, ϑ_m_, was derived for all experiments with kinetic fitting of the Langmuir adsorption isotherm. All parameters estimated from kinetic fitting are presented with their associated 95% confidence interval derived from the covariance matrix of the fit. For clarity, the ϑ_m_value of the Fab assay is written ϑ_m Fab_ and the ϑ_m_value of the Fc assay similarly ϑ_m Fc_. The ϑ_m_value of the antigen assay is termed ϑ_m antigen_. To minimize experimental variation, all antibody samples were tested in duplicate and in random sequence on a single multiplex array of assay surfaces. The coefficient of variation (CV) of ϑ_m Fc_ for the NISTmAb was 7%.

The LC-MS analysis was conducted using an HPLC-Chip Cube system coupled to a 6520 quadrupole time-of-flight (QTOF) mass spectrometer (Agilent Technologies, Santa Clara, CA) operated in electrospray ionization (ESI) positive-ion mode. Liquid chromatography was performed using a Protein-Chip (II) with a 40 nL enrichment column and analytical column of 43 mm × 75 μM with Zorbax 300SB-C8 packing material at 5 μm (G4240-63001, Agilent, Santa Clara, CA). The ChipCube source was operated at 365 °C with 5 L/min nitrogen drying gas, the capillary voltage was set to 2100 V and fragmentor at 400 V. The TOF MS acquisition range was from 100 to 4000 mass-to-charge ratio (*m*/*z*) at 13586 transients per summed spectra. The source was interfaced with an Agilent 1260/1200 series HPLC system consisting of a 1260 Cap pump, 1200 Nano pump, 1200 Micro WPS, and 1290 Infinity Thermostat (Agilent). Between 0.2 and 1 μL sample, without dilution or additional preparation, was injected onto the enrichment column using the capillary pump flow with H_2_O + 0.1% formic acid (FA) at a flow rate of 4 μL/min. The sample was eluted onto the analytical column at a flow rate of 0.6 μL/min. Solvent A and B consisted of H_2_O + 0.1% formic acid (FA) and ACN:H2O 90:10 with 0.1% FA respectively. Gradient steps are as follows: 0–4 min from 3% B to 50% B, 4–5 min to 100% B, 5–11 min 100% B, 11–12 min from 100% to 3% B. Internal lock mass calibration was performed using one calibration reference mass at 1221.9906 *m*/*z*. Data processing was performed using the Masshunter Workstation software version B.50.00 (Agilent). The mass spectra of each antibody sample are averaged from triplicate injections derived from two independent experiments.

The ESI-QTOF experiments analysed native, intact forms of the antibodies. The raw MS data undergoes a zero-charge convolution in which the area contribution of each respective charge state is summed [23]. We expressed the degree of antibody degradation as a relative area ratio of the light or heavy chain combinations compared to intact protein in the deconvoluted spectra. As we are relating relative area ratios of the same antibody in each sample, the ionization efficiency and therefore relative abundance estimates are expected to be internally consistent.

## Results

A series of kinetic traces was recorded for each antibody sample for the Fc, Fab, and antibody-antigen assays. The data are fitted to a Langmuir model with integrated rate equations given by Eq. 1 for the association phase and Eq. 2 for the dissociation phase:

1 $$ \upvartheta (t)={\upvartheta}_m\frac{k_a\left[P\right]}{k_a\left[P\right]+{k}_d}\left(1-{e}^{-\left({k}_a\left[P\right]+{k}_d\right)t}\right) $$

2 $$ \upvartheta (t)={\upvartheta}_a{e}^{-{k}_dt} $$

where ϑ(*t*) is the sensor response at time, *t*, ϑ_*m*_ as before, ϑ_*a*_ is the sensor response at the start of the dissociation phase, *k*_a_ is the association rate constant, *k*_d_ is the dissociation rate constant, and [*P*] is the concentration of the protein in solution whether Fc, Fab, or antigen. All of the parameters in Eqs. 1 and 2 are highly correlated when fitted to the data of a single concentration. However, the binding rate constants *k*_a_ and *k*_d_ may be determined by fitting the model to multiple, low-concentration samples simultaneously [19]. Furthermore, the ϑ_m_ can be determined from replicates of a high concentration sample (100 nM) binding over a time sufficient to achieve near-maximum surface coverage. Simultaneous low-concertation kinetic trace fitting and independent determination of ϑ_m_ give the most accurate determination of the interaction parameters in the Langmuir model.

**Fig. 2:**
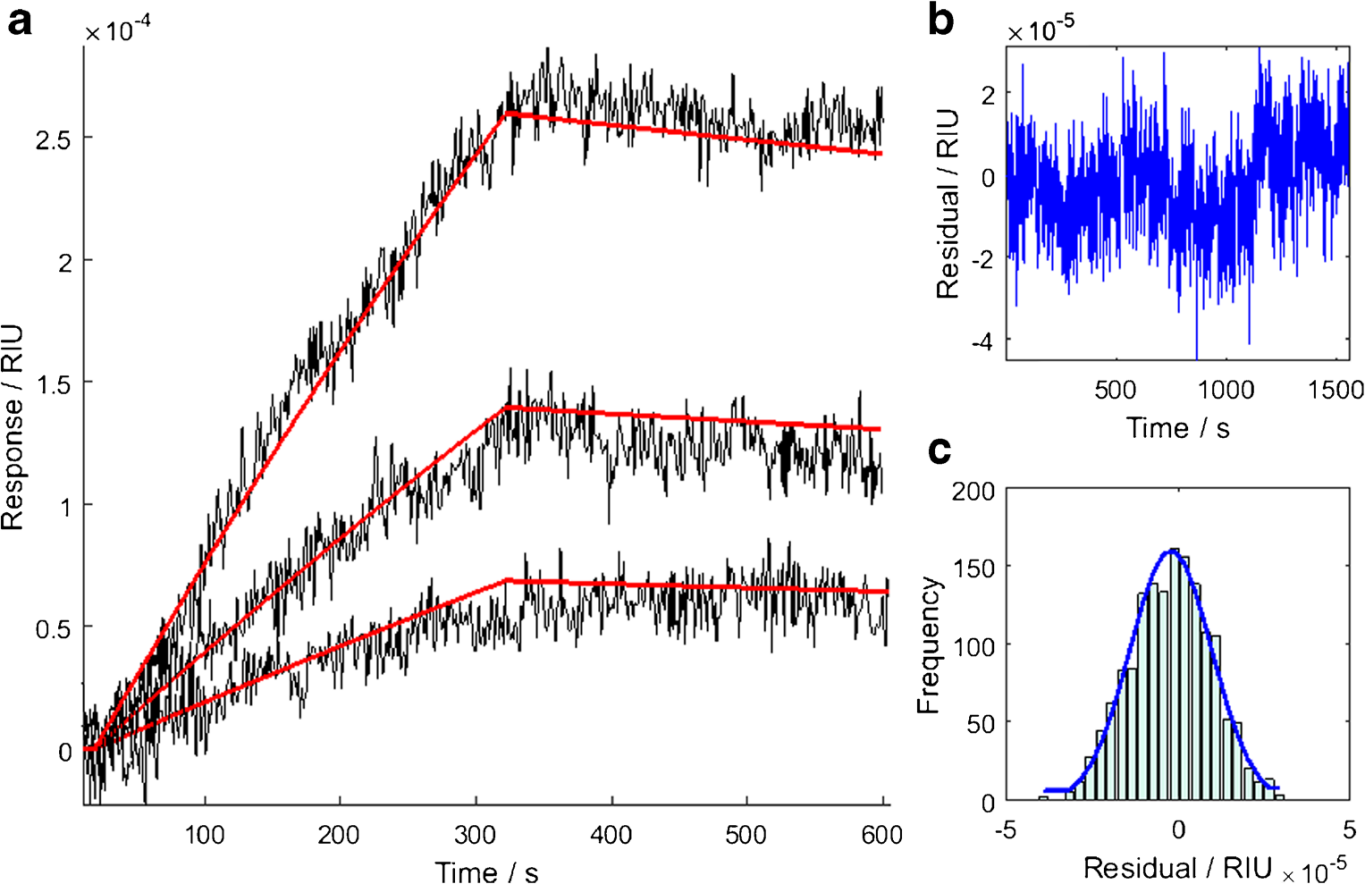
RI time dependence for the NISTmAb binding to its antigen, RVSF, immobilized on the sensor surface: **a** kinetic response curves obtained from 1.56, 3.23, and 6.25 (± 5%) nM NISTmAb samples (grey), and global fit to the Langmuir adsorption model (red); **b** residuals over the time course of the fit, showing consistent error between model fit and data; **c** histogram of the residuals indicating the error between the model fit and data is normally distributed.

The ϑ_m_ of the NISTmAb sample binding via the Fab region to its antigen (RSVF) immobilized on the sensor surface was derived as 1.73 (± 0.03) mRIU by fitting the Langmuir model to data from 100 nM antibody samples. The kinetic parameters for the NISTmAb–RSVF interaction were derived as *k*_a_ = (8.9 ± 0.2) × 10^4^ M^−1^ s^−1^, *k*_d_ = (2.4 ± 0.6) × 10^−4^ s^−1^, and *K*_D_ = 2.7 ± 0.7 nM by fitting the Langmuir model to 3 low-concentration samples (Fig. 2). The uncertainties are derived from the covariance matrix of the fit and are stated as 95% confidence intervals.

The ϑ_m_ of the NISTmAb sample binding to PAG on the sensor surface was derived as 6.7 ± 1.0 mRIU by fitting the Langmuir model to data from 100 nM antibody samples. Deviations from 1:1 Langmuir kinetics were evident in the data however a global fit to four low concentrations, whilst enabling the ϑ_m_ value to float, enables the model to fit well and estimates the kinetics of the interaction as *k*_a_ = (5.9 ± 1.1) × 10^4^ M^−1^ s^−1^ and *k*_d_ = (2.0 ± 1.9) × 10^−5^ s^−1^. The error associated with the dissociation rate constant is large due to the apparently slow dissociation rate.

**Fig. 3:**
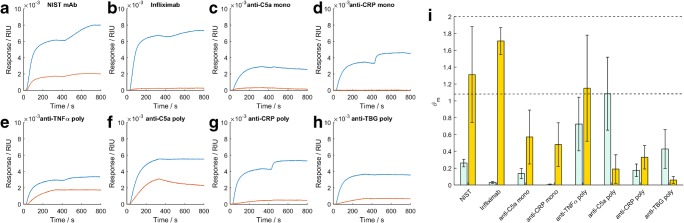
Biosensor analysis of antibody panel samples: **a**–**h** raw kinetic response of the PAG surface (blue) and antigen surface (orange) to antibody binding (0–300 s) and antigen binding (400–700 s). **i** ϑ_m_ analysis showing the ϑ_m Fab_/ϑ_m Fc_ ratio (green) and the ϑ_m antigen_/ϑ_m Fc_ ratio (gold). The ϑ_m_ values of the ϑ_m antigen_/ϑ_m Fc_ ratio are normalized for the mass of the antigen and intact antibody derived from the ESI data. Error bars are 95% confidence intervals*.* The stoichiometric limit of the normalized ϑ_m antigen_/ϑ_m Fc_ ratio is between 1 and 2 (dashed lines).

The ϑ_*m Fab*_, ϑ_m Fc_, and ϑ_m antigen_ of all antibody samples and their analytes binding to the Fab, Fc, and antigen assays were estimated from binding data (Fig. 3) using the Langmuir fitting routine and the values are listed in Table 1. All ϑ_*m *_estimates assume antibody concentrations stated in the product data sheets which were confirmed as accurate by absorbance at A280 nm. The ϑ_m Fab_ is consistently lower than the ϑ_m Fc_ for all antibody samples tested, indicating that for a given surface area, more antibodies are adsorbed when bound via the Fc region to the PAG surface compared to the antigen surface. The therapeutic infliximab biosimilar antibody has a particularly low ϑ_m Fab_ value indicating a low antigen binding density or a low epitope presentation on the sensor surface.

**Table 1:** Experimental properties of the antibody panel. All errors are 95% confidence intervals

Sample	ϑ_m Fab_/mRIU	ϑ_m Fc_/mRIU	ϑ_m antigen_/mRIU	Antigen mass/kDa	ESI mass, M_ESI_/kDa	ESI monomeric purity/%	Plasmonic Fc areal density, *M*_adsorbed_/g cm^−2^ × 10^−8^	Plasmonic Fc mass, *M*_*Fc*_/kDa	Plasmonic monomeric purity/%
NISTmAB	1.73 ± 0.03	6.66 ± 0.96	3.37 ± 0.77	53	145	94	3.66 ± 0.53	148 ± 21	100 ± 14
Infliximab biosimilar	0.22 ± 0.06	6.82 ± 0.21	1.32 ± 0.01	17	146	95	3.8 ± 0.1	152 ± 5	103 ± 3
Anti-C5a mono	0.42 ± 0.13	3.07 ± 0.28	0.11 ± 0.04	10.4	142	89	1.7 ± 0.2	68 ± 6	46 ± 4
Anti-CRP mono	0.02 ± 0.02	3.57 ± 0.13	1.44 ± 0.68	125	141	87	1.96 ± 0.07	80 ± 3	54 ± 2
Anti-TNFα poly	2.34 ± 0.13	3.23 ± 0.88	0.42 ± 0.06	17	123	61	1.8 ± 0.4	72 ± 19	49 ± 13
Anti-C5a poly	5.82 ± 1.48	5.36 ± 0.60	0.06 ± 0.04	10.4	127	65	3.0 ± 0.3	119 ± 12	80 ± 8
Anti-CRP poly	0.76 ± 0.30	4.33 ± 0.09	1.18 ± 0.45	125	132	72	2.38 ± 0.05	96 ± 2	65 ± 1
Anti-TBG poly	1.60 ± 0.70	3.74 ± 0.24	0.08 ± 0.05	54	119	54	2.1 ± 0.1	83 ± 5	56 ± 3

An estimate of the relative binding site density differences between PAG and the antigens may be obtained by comparing the ϑ_m Fab_/ϑ_m Fc_ ratio of the NISTmAb (0.26 ± 0.04) to that of the panel of antibodies with a range = (0.004–1.1) × 10^−3^. Furthermore, the ϑ_m antigen_/ϑ_m Fc_ ratio of the antibody samples is an estimate of antibody monomeric purity (related to moles/moles), after normalizing for molecular mass by dividing ϑ_m antigen_ and ϑ_m Fc_ by the antigen masses and intact IgG masses respectively (Fig. 3i).

**Fig. 4:**
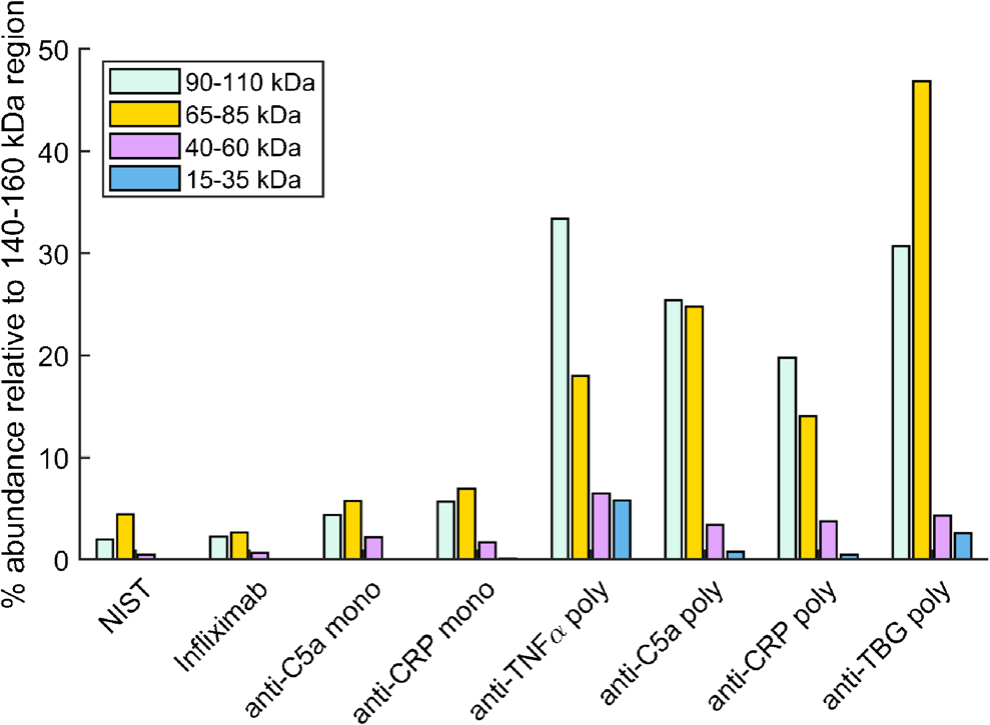
The abundances of fragments in each antibody sample evaluated by taking the AUC of specific regions of the mass spectra corresponding to IgG fragmentation products. The percentage abundances of the most abundant peaks are plotted relative to the AUC of the intact region (140–160 kDa): 90–110 kDa (light blue), 65–85 kDa (gold), 40–60 kDa (pink), and 15–35 kDa (blue). All regions may contain Fc moieties.

To validate the ϑ_m antigen_/ϑ_m Fc_ ratio as a measure of antibody monomeric purity, the biosensor results were compared with results from the ESI mass spectrum recorded for each antibody (see Electronic Supplementary Material (ESM) Fig. S1). The deconvolved mass spectrum of the NISTmAb is dominated by a species at 148.2 kDa attributed to the pure, non-fragmented IgG structure. The NISTmAb Fab fragment has a reported mass of 47 (± 5) kDa [16] and the mass spectrum recorded here shows a low intensity mass peak at 48 kDa. All antibody samples show additional lower mass proteins with varying abundances attributed to any of the fragments shown in Fig. 1, including dissociated heavy chain (Hc) and light chain (Lc). The relative abundances of fragments relative to the intact antibody (Fig. 4) are determined from the area-under-the-curve (AUC) of regions in which masses are typically present: 140–160 kDa, 90–110 kDa, 65–85 kDa, 40–60 kDa, and 15–35 kDa. These regions of the mass spectrum correspond to the predicted fragmentation products shown in Fig. 1.

The HcLc fragment, expected at 75 kDa, is the most abundant fragmentation product in all monoclonal antibody samples, whilst the Fab-Fc fragment is most abundant in the polyclonal samples, with the exception of anti-TBG. The AUC of impurities relative to the intact IgG AUC is < 20% for monoclonal antibodies. In contrast, all polyclonal samples display impurities present at > 20% relative to the intact IgG structure. The monomeric purity estimated by ESI is the peak area of the intact region expressed as a percentage of total peak area (area/area) and is tabulated in Table 1, expressed as a mean of 3 mass spectra and a 95% confidence limit of ± 2%. The monomeric purity of the NISTmAb is determined by ESI to be 94 ± 2% and compares well to the reported value 97% by NIST [8].

## Discussion

Antibody monomeric purity is critical for immunoassay development, binding kinetics studies, and effective immunotherapy. The Fab assay provides data for kinetic analysis of the antibody-antigen binding reaction, whilst the Fc and antigen assays provide potential assays for the rapid assessment of antibody monomeric purity. The Fc assay may identify low monomeric purity based on a potential mass deficit observed for fragmented samples binding to a constant number of binding sites on the PAG surface. The antigen assay assesses monomeric purity using the binding ratio of antigen to antibody captured by the PAG surface. Results are validated using monomeric purity estimates from native ESI-QTOF mass spectrometry.

ESI data suggest the most abundant glycoform of the intact NISTmAb has a mean molecular mass of 148.2 kDa and the sample displays a distinctive glycosylation pattern in good agreement with published values [11]. The homogeneity of the NISTmAb contrasts with the heterogeneity of intact IgG masses in the anti-C5a monoclonal, anti-CRP monoclonal, and anti-CRP polyclonal samples, attributed to multiple glycoforms (ESM Fig. S2) [24]. The ESI data validate the NISTmAb as a good standard antibody of high monomeric purity. From the ESI mass spectrum data, the mean mass of proteins in the antibody samples, *M*_ESI_, can be derived:

3 $$ {M}_{ESI}=\frac{aA+ bB+ cC+ dD}{a+b+\mathrm{c}+\mathrm{d}} $$

where lowercase letters represent the percentage AUC of the mass spectrum relative to the intact peak area (140–160 kDa) from the regions: 90–110 kDa (predicted Fab-Fc complex), 65–85 kDa (predicted HcLc complex), 40–60 kDa (predicted Hc or Lc), and 15–35 kDa (predicted Lc or Hc monomer of Fc). Uppercase letters represent the mean mass of the integration region. The derived *M*_ESI_ values of each sample in the panel are shown in Table 1.

The monomeric purity of the NISTmAb and the therapeutic infliximab biosimilar is high, which is not the case for the other commercially available samples that would be used for research applications. The reliability of experiments involving Western blots, immunohistochemistry, flow cytometry, and immunoprecipitation is negatively impacted by variations in antibody purity as these techniques fundamentally rely on a connection between Fc and Fab regions of the antibody. The consequence of purity on kinetic analysis also needs to be considered with the fundamental errors of the 1:1 binding model or Langmuirian kinetics conventionally used to analyse antibody binding events. Fitting a set of low concentrations in the global fit methodology typically produces an error of 15% for *k*_a_ and 10% for *k*_d_ over the timescale of the experiments [19]. Model fitting errors would dominate the analysis of the NISTmAb and infliximab biosimilar samples but this would not be true for all members of the antibody panel tested here. For the case of the anti-TBG sample, any reported antibody kinetics analyses without a sample purity assessment and correction may have an error of up to 45% due to low monomeric purity.

The packing density of IgG around a protein is a property of epitope density, antibody conformation, and monomeric purity. Antibodies with identical masses should achieve identical ϑ_m_ values for a given antibody binding density, albeit after differing periods of time to allow variations in association kinetics. There is significant variation in the ϑ_m Fab_ values recorded (Table 1) which may be explained by variation in availability of epitopes for antibody binding as evidenced by the larger ϑ_m Fab_ of polyclonal anti-CRP compared to the monoclonal anti-CRP sample on the same CRP surface. In contrast, the ϑ_m Fc_ shows less variation than ϑ_m Fab_, attributed to the use of a common PAG surface for all antibody samples with a consistent number of binding sites.

Variation in ϑ_m Fc_ may occur due to differing mean IgG mass differences (monomeric purity differences) between IgG samples as observed with ESI. The Fc assay presented here will bind with high affinity to both intact IgG and the most abundant fragmentation products which contain the Fc region (Fig. 1), a concern for PAG-based affinity purification of antibodies but a potential antibody fragmentation assay when combined with surface plasmon technology. Any given ϑ_m_ derived from fitting of the Langmuir equation (Eqs. 1 and 2) may be related to the adsorbed mass on the surface, *M*_*adsorbed*_, by de Feijter’s formula [25]:

4 $$ {M}_{\mathrm{adsorbed}}={d}_A\frac{\Delta  RI}{\raisebox{1ex}{$ dn$}\!\left/ \!\raisebox{-1ex}{$ dc$}\right.} $$

where *d*_A_ is the thickness of the adsorbed layer, *dn/dc* is the rate of change of refractive index with surface concentration (the refractive index increment), and ΔRI is the change in the refractive index recorded. The value of *dn/dc* has been measured for protein as 0.182 g cm^−3^ and has been shown to vary with buffer conditions [26] but the effect is small [25].

The largest uncertainty in the estimate of *M*_adsorbed_ is the value of *d*_A_. IgG layers adsorbed onto protein A and silica surfaces have been observed previously using ellipsometry with thicknesses of 4 nm [27] to 16 nm [28] respectively. Neutron reflectivity measurements of IgG4 binding to a protein A/BSA surface report three sequential layers leading to total thicknesses between 6.1 nm and 25 nm depending on washing and blocking of the surface [29] because non-specific 3D aggregation at high IgG concentrations can enable adsorbed layers to exceed the maximum length of a single IgG molecule [30]. The mean cross-section dimension of the IgG crystal structure [31] suggests a *d*_A_ of 10 nm, assumed constant for all antibody samples here. The derived surface adsorbed mass density therefore depends only on the difference in the refractive index (∆RI) of the adsorbed antibody.

Substituting ϑ_m Fc_ of NISTmAb for ∆RI in Eq. 4 predicts the areal density of the NISTmAb on the PAG surface, *M*_adsorbed_ = 3.7 ± 0.5 × 10^−8^ g cm^−2^. By comparison, electrostatic adsorption of monoclonal antibodies onto a variety of surfaces has been reported at densities in the range 5 × 10^−7^ to 5 × 10^−6^ g cm^−2^ [32, 33]. Electrostatic adsorption is expected to achieve a higher surface mass density compared with the ordered two-dimensional layer of IgG bound to a PAG surface. The *M*_adsorbed_ values of each antibody studied are presented in Table 1.

The ESI data indicate a high monomeric purity in the NISTmAb sample (94% ± 2) with an intact mass, *M*_monomer_, of 148.2 kDa. The NISTmAb data and the Avogadro constant, *N*_A_, can be used to calibrate the surface density of antibodies bound at saturation, *N*_Ab_ = *M*_adsorbed_ × *N*_A_/*M*_monomer_ = (1.49 ± 0.21) × 10^11^ cm^−2^. Comparatively, the maximum number of monoclonal antibodies in a monolayer has been determined by adsorption onto a silicon nitride surface as 1.2 × 10^12^ cm^−2^ [34]. The unknown purity of antibody samples in the literature is a potentially large source of error for published *N*_Ab_ values derived from protein mass density measurements. The *N*_Ab_ figure presented here is of known accuracy due to the monomeric purity estimate from ESI.

The number of PAG binding sites on the surface is constant for NISTmAb and if all antibodies bind to the surface with the same packing density then an estimate of effective mass at each binding site may be estimated from a simple ratio:

5 $$ {\upvartheta}_m^{Fc}={\upvartheta}_m^{\mathrm{monomer}}\times \frac{M_{Fc}}{M_{\mathrm{monomer}}} $$

where ${\upvartheta}_m^{\mathrm{monomer}}$ and *M*_monomer_ are the ϑ_m Fc_ and mass of the monomeric reference material, which in the present study is the NISTmAb with ϑ_m Fc_ = 6.66 ± 0.96 mRIU and a mass of 148.2 kDa. When an antibody sample has degraded, a fraction of Fc binding domains on the PAG will be occupied by antibody fragments with a lower mean molecular mass than the intact IgG reference and will exhibit a lower ϑ_m Fc_ for a constant number of occupied binding sites. The Fc assay can be used to estimate monomeric purity by expressing *M*_Fc_ as a percentage of *M*_monomer_ (Table 1).

A weak positive correlation is observed between ϑ_m Fc_ and ESI monomeric purity and several phenomena may account for the variations observed in ϑ_m Fc_. Firstly, *N*_Ab_ at ϑ_m Fc_ may be a fundamental property of a given antibody due to glycan variation and the locations of PAG binding domains on the structure, leading to some antibody monomers binding in orientations which favour closer packing on the surface [35]. The ϑ_m Fc_ will also be sensitive to non-specific aggregation and clustering of IgG [29], although aggregates of the NISTmAb IgG are only observed following deliberate physical agitation for days at room temperature [36] and the antibody samples tested here are not expected to contain significant aggregates due to appropriate storage. The lack of correlation between ϑ_m Fc_ and ESI monomeric purity may best be explained by the orientation and higher packing density of low mass antibody degradation products, which may have access to a greater number of binding sites on the PAG surface than monomeric IgG [20]. Therefore, it is likely that the ϑ_m Fc_ of the Fc assay is only sensitive to severe antibody degradation.

**Fig. 5:**
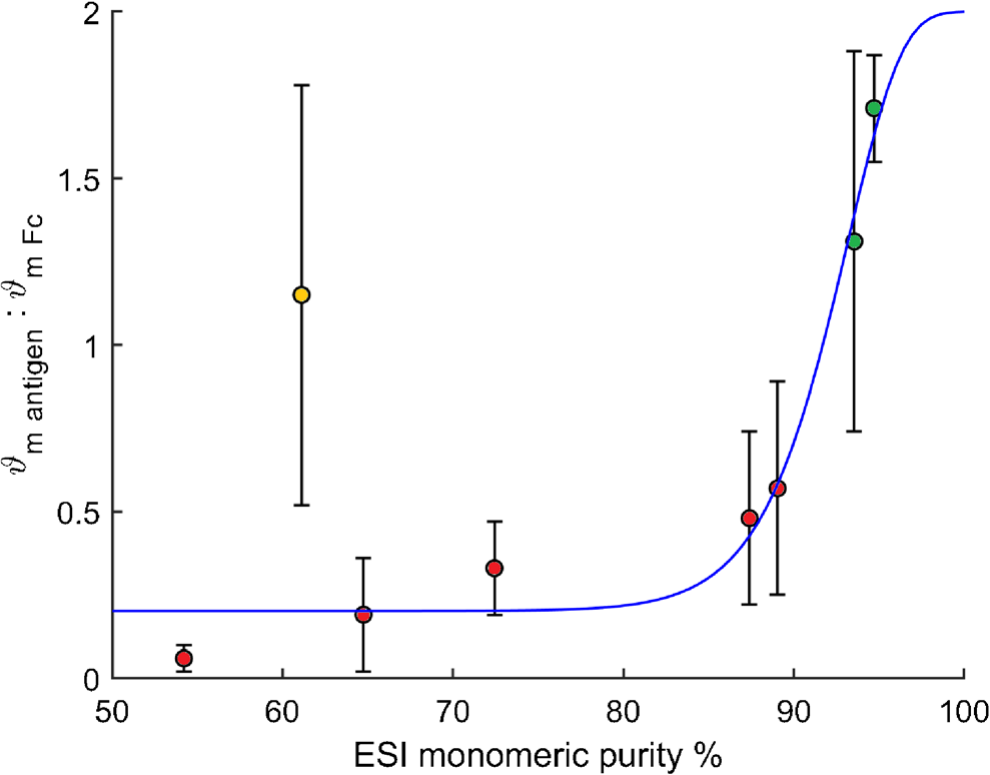
The relationship between monomeric purity estimated from the ESI data and the mass -normalized ϑ_m antigen_/ϑ_m Fc_ biosensor measurement. The NISTmAb and infliximab biosimilar (green) are > 90% pure according to ESI data and exhibit ϑ_m antigen_/ϑ_m Fc_ ratios within the expected limits of between 1 and 2 antigens per antibody. Other commercially available antibodies (red) are < 90% pure and show less than 1 antigen bound per antibody. A 5-parameter logistic curve fits well to the data (blue) and is constrained by a maximum asymptote of 2 (the upper limit of the antigen-antibody interaction stoichiometry). The anti-TNFα polyclonal (orange) does not fit the trend line, likely due to a unique fragmentation pattern resulting in a majority of 100 kDa HcLc fragments which may pack tightly on the PAG surface and remain capable of binding 1 antigen

Our results show that a more sensitive measure of monomeric purity is to support antibodies on the PAG surface and measure the antigen binding ratio, ϑ_m antigen_/ϑ_m Fc_, which after normalizing for molecular mass, shows a strong dependence on monomeric purity estimated by ESI (Fig. 5).

The infliximab biosimilar antibody binds to a relatively low mass antigen (TNFα with a monomer mass of 17 kDa) and displays an antigen-antibody binding ratio of 1.71 ± 0.16. The NISTmAb binds to a higher-mass antigen (53 kDa) and displays a lower antigen-antibody binding ratio of 1.31 ± 0.57 attributed to greater steric hindrance of antigens. The ϑ_m antigen/_ϑ_m Fc_ ratio threshold to determine monomeric purity of antibody samples is antigen-dependent for larger antigens but would always exceed 1 with adequate antibody spacing on a plasmonic surface. The NISTmAb performs well as a standard material for the antibodies shown, but calibration of the assay would likely be improved by comparing each tested sample to a high-purity reference standard which binds to a common antigen.

Of the commercially available research antibodies, anti-TBG antibody shows a relatively high ϑ_m Fab/_ϑ_m Fc_ ratio but displays the weakest ϑ_m antigen_/ϑ_m Fc_ ratio. These two ratios indicate that both Fab and Fc regions of TBG are present in the sample, but a significant proportion is separated due to degradation. The high fragmentation of the anti-TBG antibody is supported by the lack of intact antibody in the ESI mass spectrum (ESM Fig. S1).

The ϑ_m antigen_/ϑ_m Fc_ ratio is a measure of the biological activity of an antibody sample, whereas the ESI monomeric purity estimated from mean mass penalizes any form of antibody fragmentation. The ESI data predict a low monomeric purity in the anti-TNFα polyclonal but the sample displays a relatively high ϑ_m antigen_/ϑ_m Fc_ ratio. The result may be explained by the unique degradation pattern shown in Fig. 4 which indicates that the most abundant species is the 100 kDa HcLc fragment which is capable of binding to PAG and one antigen. The fragmentation in the anti-TNFα polyclonal remains identifiable by comparison to the infliximab biosimilar which shares the same antigen but exhibits a higher antigen-antibody binding ratio.

Using the ϑ_m Fab/_ϑ_m Fc_ ratio recorded by the biosensor, the antibody panel readily divides into two types of samples: the high-purity (> 90%) samples (NISTmAb and infliximab biosimilar) and the remainder of the panel with lower monomeric purity (< 90%). The ϑ_m Fab/_ϑ_m Fc_ ratio is significantly different for these two material types as evidenced by a two-sample *t* test and two-sample Kolmogorov-Smirnov (*P* = 0.01 and *P* = 0.03 respectively). The assays developed here show the potential to test the specification of antibodies immediately before use as assay materials and in other antibody-dependent techniques to prevent variability in experimental results caused by sample degradation. Both the ESI and biosensor-based assays provide an estimate of monomeric purity in 15 min but the biosensor platform exhibits some key advantages. The biological activity of therapeutic antibody at both the Fab and Fc regions is critical and can be tested on the small form factor multiplexed biosensor platform when reliably calibrated using the reference NISTmAb.

## Conclusions

The NISTmAb provides an important new benchmark material which enables the calibration of biosensors assessing monomeric purity of antibody samples with assays for Fab, Fc, and antigen. The analysis of a panel of commercially available research grade antibodies, using ESI and biosensor surfaces, highlights significant variation in monomeric purity and raises accuracy concerns for previously reported *k*_a_, *k*_d_, and *K*_D_ measurements of similar materials in the literature and the risk to reproducibility. Without sample monomeric purity analysis (of both the antibody and the antigen) these measurements may have errors approaching 50% in highly fragmented samples. The biosensor-based technique presented here to estimate antibody monomeric purity can be performed in 15 min and may be combined with additional rapid tests for IgG aggregates as described for the NISTmAb in ref. [36].

## Electronic supplementary material

ESM 1 (PDF 428 kb)
