# Supplementary material for: A rapid and quantitative technique for assessing IgG monomeric purity, calibrated with the NISTmAb reference material
Source: Anal Bioanal Chem. 2019 Aug 2;411(24):6487–96. doi: 10.1007/s00216-019-02029-0 (PMC6718376; doi:10.1007/s00216-019-02029-0)
Supplement: Supplementary file 1 — (PDF 428 kb) [file 216_2019_2029_MOESM1_ESM.pdf]

Analytical and Bioanalytical Chemistry

Electronic Supplementary Material

A rapid and quantitative technique for assessing IgG monomeric purity, calibrated with the NISTmAb reference material

Peter P. Reader, Rouslan V. Olkhov, Shaun Reeksting, Anneke Lubben, Christopher J. Hyde, Andrew M. Shaw

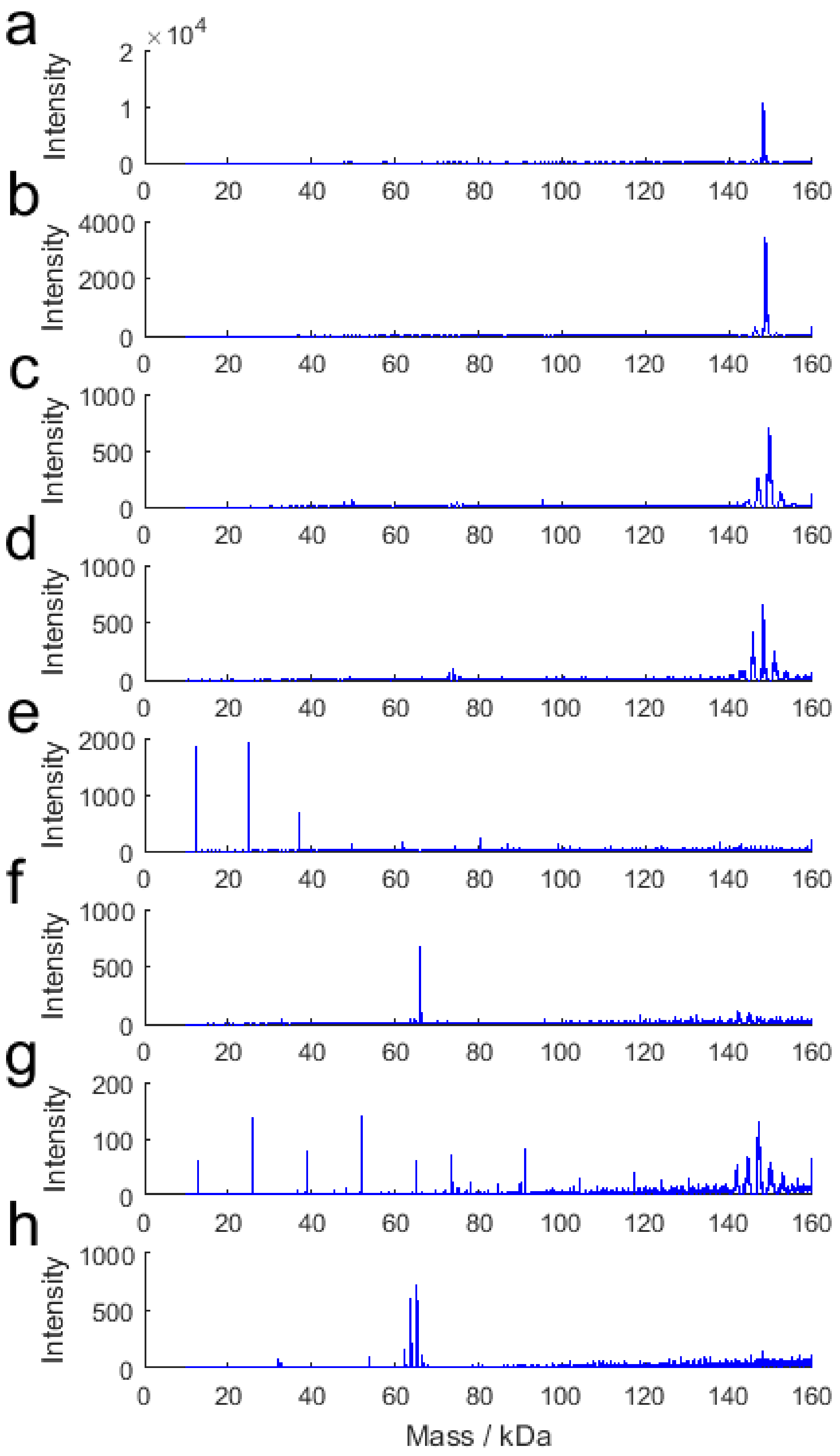

Fig. S1 The deconvolved mass spectra of 8 antibody samples: a) the NIST RM antibody; b) Infliximab biosimilar; c) anti-C5a monoclonal; d) anti-CRP monoclonal; e) anti-TNFα polyclonal; f) anti-C5a polyclonal; g) anti-CRP polyclonal and h) anti-TBG polyclonal. All samples show varying intensities of intact IgG at ~150 kDa, often with multiple glycoforms. All samples with the exception of NIST RM contain fragmented antibody components: single heavy chain – light chain fragment (~75 kDa), single heavy chain (~50 kDa) and single light chain (~25 kDa)

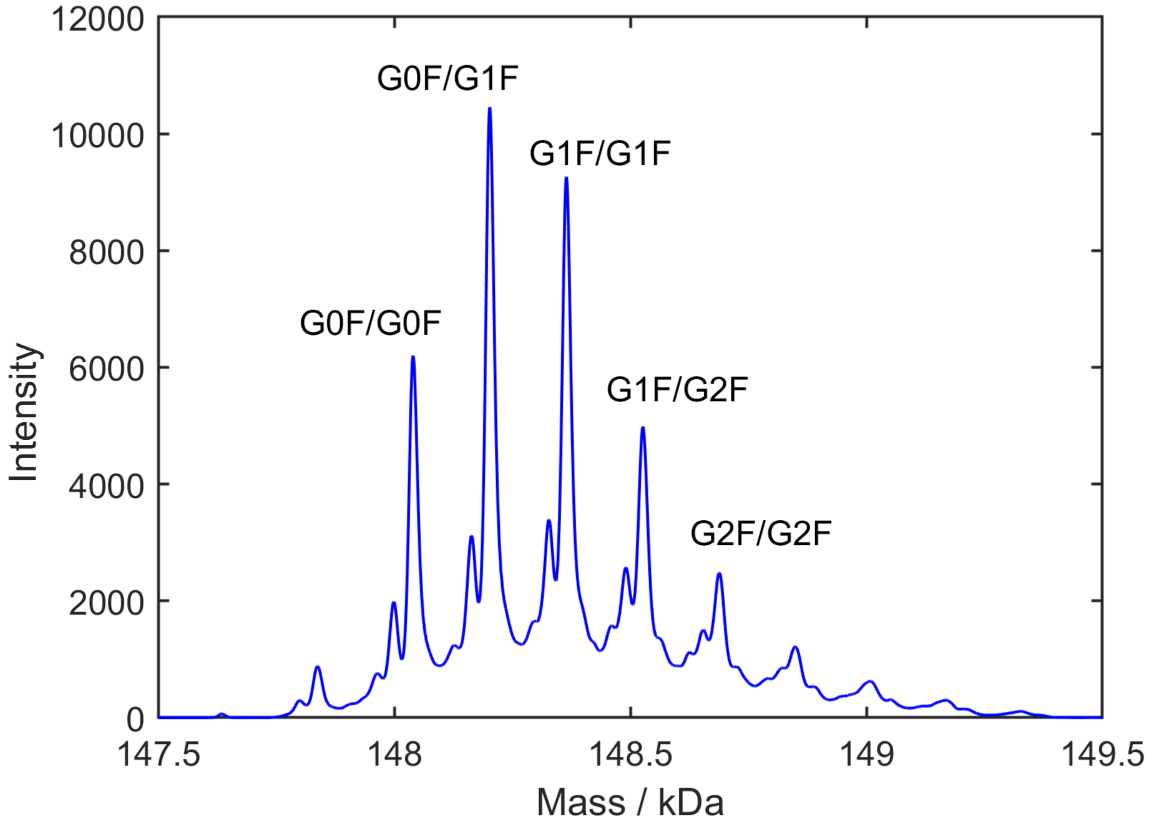

Fig. S2 Mass spectrum of the NIST RM intact IgG region. The highest intensity peak has a mass of 148.2 kDa and the glycosylation pattern is in good agreement with previously identified glycoforms in mass spectrometry studies
